# Naphthoquinone Derivatives as Scaffold to Develop New Drugs for Tuberculosis Treatment

**DOI:** 10.3389/fmicb.2018.00673

**Published:** 2018-04-09

**Authors:** Priscila C. B. Halicki, Laís A. Ferreira, Kelly C. G. De Moura, Paula F. Carneiro, Karina P. Del Rio, Tatiane dos S. C. Carvalho, Maria do C. F. R. Pinto, Pedro E. A. da Silva, Daniela F. Ramos

**Affiliations:** ^1^Núcleo de Pesquisa em Microbiologia Médica, Faculdade de Medicina, Universidade Federal do Rio Grande, Rio Grande, Brazil; ^2^Núcleo de Pesquisas em Produtos Naturais, Centro de Ciências da Saúde, Universidade Federal do Rio de Janeiro, Rio de Janeiro, Brazil

**Keywords:** *Mycobacterium tuberculosis*, naphthoquinones, antimicrobials, resistance, tuberculosis

## Abstract

Despite being a curable disease, tuberculosis (TB) remains a public health problem worldwide mainly due to lengthy treatment, as well as its toxic effects, TB/HIV co-infection and the emergence of resistant *Mycobacterium tuberculosis* strains. These barriers reinforcing the need for development of new antimicrobial agents, that ideally should reduce the time of treatment and be active against susceptible and resistant strains. Quinones are compounds found in natural sources and among them, the naphthoquinones show antifungal, antiparasitic, and antimycobacterial activity. Thus, we evaluated the potential antimycobacterial activity of six 1,4-naphthoquinones derivatives. We determined the minimum inhibitory concentration (MIC) of the compounds against three *M. tuberculosis* strains: a pan-susceptible H37Rv (ATCC 27294); one mono-resistant to isoniazid (ATCC 35822); and one mono-resistant to rifampicin (ATCC 35838); the cytotoxicity in the J774A.1 (ATCC TIB-67) macrophage lineage; performed *in silico* analysis about absorption, distribution, metabolism, and excretion (ADME) and docking sites. All evaluated naphthoquinones were active against the three strains with MIC between 206.6 and 12.5 μM, and the compounds with lower MIC values have also showed low cytotoxicity. Moreover, two naphthoquinones derivatives 5 and 6 probably do not exhibit cross resistance with isoniazid and rifampicin, respectively, and regarding ADME analysis, no compound violated the Lipinski’s rule-of-five. Considering the set of findings in this study, we conclude that these naphthoquinones could be promising scaffolds to develop new therapeutic strategies to TB.

## Introduction

According to World Health Organization, tuberculosis (TB) is one of the top 10 causes of death worldwide and, in 2016, there were an estimated the incidence of 10.4 million new cases (WHO, 2017). The main strategies for reducing TB morbidity and mortality rates are related to prophylaxis, early diagnosis and effective treatment of the disease. Bacille Calmette–Guérin (BCG) vaccine has been administered since the beginning of the 20th century in the immunization process, but only protects against the most severe cases of TB (Andersen and Woodworth, 2014; Stefanova, 2014). Despite the technological advances in the diagnostic area and the absence of an effective vaccine, the therapy still is the main tool for TB control.

The first-line therapy regimen for TB is a combination of four drugs: isoniazid (INH), rifampicin (RIF), pyrazinamide and ethambutol, which cure about 85% of patients infected with susceptible strains (WHO, 2017). Other antimicrobials such as kanamycin, amikacin, capreomycin, ethionamide, and levofloxacin are considered second-line drugs. These drugs are also used in the treatment of TB, especially in cases of multidrug-resistant strains (MDR) infection – which are resistant to both INH and RIF – and when there is intolerance to first-line drugs. However, second-line therapy is more expensive, more toxic, and less effective than basic therapy (Ma et al., 2010).

Over the past 15 years, the TB treatment was able to avoid at least 50 million deaths, however, there is still a gap in this regard (WHO, 2017). Although it is a curable disease, there are several factors that hamper the TB control: lengthy treatment, toxic effects, pharmacokinetic interactions with other drugs, TB-HIV co-infection and the emergence of drug resistance (Palomino et al., 2009). Still in this context, the available therapeutic arsenal is insufficient, being ineffective against resistant strains and latent TB (Ma et al., 2010). Thus, new antimicrobial agents with novel mechanisms of action could be effective in the management of TB, reducing cross-resistance, the length of treatment and the adverse effects. It would increase the patient compliancy to the therapy, favoring the cure (Hoagland et al., 2016; Igarashi et al., 2017).

The natural sources have been highlighted in the discovery and development of new antibiotics mainly due to structural diversity of the compounds, therapeutic potential and plethoric mechanisms of action (Bérdy, 2012; Newman and Cragg, 2016). Among the compounds that may be found in a variety of plants, algae, bacteria and fungi, are quinones: aromatic compounds which are classified in anthraquinones, benzoquinones and naphthoquinones, according to their chemical compositions (López-López et al., 2014). The naphthoquinones and derivatives have a naphthalene ring, and some studies have reported its antibacterial, antitumoral, antileishmanial, anthelmintic, and antifungal activity (Coelho et al., 2010; Hazra et al., 2013; Fiorito et al., 2014; Neto et al., 2014; Mata-Santos et al., 2015). Some mechanisms of action have been proposed for naphthoquinones, mainly related to oxidative stress – due to the production of reactive oxygen species (ROS) which induce apoptosis in biological systems (Da Silva et al., 2003) – and to inhibition of DNA gyrase (Karkare et al., 2013).

Previous research has demonstrated the antimycobacterial activity of naphthoquinones (Mital et al., 2008; Coelho et al., 2010; Ferreira et al., 2010; Cantos et al., 2012; Uc-Cachón et al., 2014), including against multidrug and extensively drug resistant strains (Dey et al., 2014), bringing attention to the potential of these compounds as a basis for the development of new anti-TB drugs. In this scenario, we aimed to evaluate the antimycobacterial activity of six 1,4-naphthoquinones derivatives against susceptible and drug resistant strains of *Mycobacterium tuberculosis*.

## Materials and Methods

### Synthesis

The naphthoquinone **1** is commercial, and the other compounds were synthetized using methods previously described in the literature. Naphthoquinones **2** and **3** were synthesized via cyclization of lapachol and nor-lapachol, respectively (De Moura et al., 2001), while naphthoquinone **4** was synthesized via lawsone alilation in a two steps procedure (Pinto et al., 1997). The amination of 1,4-naphthoquinone with sodium azide resulted in the naphthoquinone **5** (Fieser and Hartwell, 1935) while the amination with aniline resulted in the naphthoquinone **6** (Lisboa et al., 2011) (**Table 1**). All compounds were solubilized in dimethyl sulfoxide 99.5% (Sigma-Aldrich) at a concentration of 10 mg/ml and stored at 4°C until used.

**Table 1:** Characterization of the 1,4-naphthoquinone derivatives.

Chemical structure		Chemical formula	Nomenclature
**1**	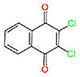	C_10_H_4_Cl_2_O_2_	2,3-Dichloronaphthalene- 1,4-dione
**2**	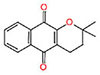	C_15_H_14_O_3_	2,2-Dimethyl-3,4-dihydro- 2H-benzo[g]chromene-5, 10-dione
**3**	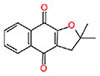	C_14_H_12_O_3_	2,2-Dimethyl-2,3-dihydronaphtho[2,3-b]furan-4,9-dione
**4**	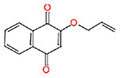	C_13_H_10_O_3_	2-(Allyloxy)naphthalene-1,4-dione
**5**	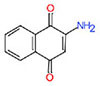	C_10_H_7_NO_2_	2-Aminonaphthalene-1,4-dione
**6**	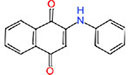	C_16_H_11_NO_2_	2-(Phenylamino)naphthalene- 1,4-dione

### Strains and Inoculum

The antimicrobial activity of naphthoquinones was evaluated against three strains of *M. tuberculosis*: a pan-susceptible, H37Rv (ATCC 27294); a mono-resistant to INH (INH_R_ – ATCC 35822) with mutation in the *katG* gene (S315T); and a mono-resistant to RIF (RMP_R_ – ATCC 35838) with mutation in *rpoB* gene (H526Y). All strains were cultured in Ogawa-Kudoh, for up to 14 days at 37°C without CO_2_. The inoculum for each strain was prepared in a glass tube containing beads to break the clumps, in sterile distilled water, according to 1.0 McFarland scale (3 × 10^8^ UFC/mL) (Woods et al., 2011). After this process, it was diluted at a ratio of 1:20 in Middlebrook 7H9 Broth. The tests were performed at the Medical Microbiology Research Center (NUPEMM), at the Federal University of Rio Grande (FURG), under strict conditions required for handling *M. tuberculosis*.

### Resazurin Microtiter Assay (REMA)

The minimum inhibitory concentration (MIC) of the compounds was determined by Resazurin Microtiter Assay (Palomino et al., 2002). Briefly, in a 96-well microplate, 100 μl of 7H9 medium supplemented with 10% OADC (Oleic acid Albumin Dextrose Catalase) was added in the test wells. In the first line was added 100 μl of each compound previously diluted in sterile water; a serial microdilution (1:2) was performed in the x-axis and, after, 100 μl of the last well was discarded. At the end of microdilution was added 100 μL of inoculum in each well and the final range of concentrations evaluated was 200 to 0.8 μg/mL for naphthoquinones, 8 to 0.03 μg/mL for INH and 1.024 to 0.06 μg/mL for RIF. In each plate were added sterility and growth controls. After incubation at 37°C for 7 days was added 30 μL of resazurin 0.02% on the plate and then, 24 h later, the reading of the results was performed. MIC was defined as the lowest concentration of compound capable of inhibiting bacterial growth. All tests were performed in triplicate.

### Cytotoxicity Assay

The cytotoxicity of the compounds was evaluated on adherent J774A.1 macrophage cell line (ATCC TIB-67). In a 96-well plate, 200 μL of cell suspension (at the concentration of 1 × 10^5^ cells/mL) were cultivated in Dulbecco’s modified Eagle medium (Sigma-Aldrich) supplemented with 10% of fetal bovine serum and maintained for 24 h at 37°C in a humid atmosphere with 5% CO_2_ (Snewin et al., 1999). After this period, the attached cells were exposed to different concentrations of naphthoquinones (200 to 0.8 μg/mL) and the plate was again incubated overnight. To determinate the concentration of the compound capable of maintaining the viability of 50% of the cells (IC_50_) were added 30 μL of resazurin 0.01% and after 24 h of incubation the fluorescence was measured by Thermo Plate TP-Reader BioTek^TM^ ELx800^TM^ (Ansar Ahmed et al., 1994).

### Selectivity Index (SI)

The SI of the compounds was calculated based on the results of MIC and IC_50_ of each compound for each strain, according to the following formula: *IC_50_*/*MIC*. SI values equal to or greater than 10 indicate that the compound is pharmacologically safe (Orme, 2001; Pavan et al., 2012).

### Theoretical Properties of Absorption, Distribution, Metabolism, and Excretion (ADME)

Characteristics of theoretical absorption, distribution, metabolism, and excretion (ADME) and toxicological effects of the compounds were determined by i*n silico* analysis, using the free software: Molinspiration^1^, Swiss ADME^2^ (Daina et al., 2017), and OSIRIS Property Explorer^3^. According to the Lipinski Rule-of-Five, the following physicochemical parameters were evaluated: molecular weight, logP, H-bond donors, and H-bond acceptors (Lipinski, 2004).

### Docking Analysis

Flexible docking simulation was performed by ArgusLab 4.0.1, using *Escherichia coli* RNA polymerase as a protein template. The structures were from Protein Data Bank (PDB)^4^ – files 5UAC and 5UAQ. The interaction between proteins (wild-type and mutant H526Y) and the ligands (RMP and compound **6**) was evaluated from residues 507 to 533, which comprise the RMP resistance-determining region (RRDR) (Ramaswamy and Musser, 1998). In the docking calculations, it was applied the Ascore as scoring method (Luo et al., 2012).

## Results

All naphthoquinones showed inhibitory activity against the three *M. tuberculosis* strains with MIC ranging between 206.6 and 12.5 μM (**Table 2**). Besides the naphthoquinones being active against the susceptible strain, the compounds also showed various levels of activity against the resistant strains (**Table 2**). The compounds **1** and **3** showed, respectively, MIC = 110.6 and 54.8 μM, for all strains evaluated, while naphthoquinones **2** and **4** showed lower inhibitory concentrations against the susceptible strain, compared to the resistant strains. In addition, compounds **1**, **2**, and **4** exhibited IC_50_ between 103 and 285 μM, resulting in SI values between 0.07 and 2.8.

**Table 2:** Activity of naphthoquinones against three *M. tuberculosis* strains and IC_50_ on J774A.1 cells lineage.

Compound	MIC (μM)			IC_50_ (μM)	SI (*IC_50_*/*MIC*)		
	H37Rv	INH_R_	RMP_R_	J774A.1	H37Rv	INH_R_	RMP_R_
**1**	110.6	110.6	110.6	103	0.9	0.9	0.9
**2**	103.3	206.6	206.6	285	2.8	1.4	1.4
**3**	54.8	54.8	54.8	>877	≥16	≥16	≥16
**4**	58.4	234	234	173	0.3	0.07	0.07
**5**	72.2	36.1	36.1	>1156	≥16	≥32	≥32
**6**	100.4	100.4	12.5	>803	≥8	≥8	≥64
INH	0.438	14.6	≤0.219	3733	8523	256	ND
RIF	0.608	0.304	622.2	>2489	≥4094	≥8187	≥4

*MIC, minimum inhibitory concentration; IC50, inhibitory concentration of 50%; SI, Selectivity Index; INHr, ATCC 35822; RMPr, ATCC 35838; ND, not determined.*

For the INH_R_ strain, compared with the H37Rv, all compounds showed equal or reduced activity, except for compound **5**. On the other hand, in comparison with the RMP_R_ strain, the only difference in MIC was related to the compound **6**. Interestingly, the compound **6** was the most active against RMP_R_ strain (MIC = 12.5 μM), in a concentration eight times less than the concentration capable of inhibiting the growth of H37Rv and INH_R_ strains (MIC = 110.4 μM).

**FIGURE 1:**
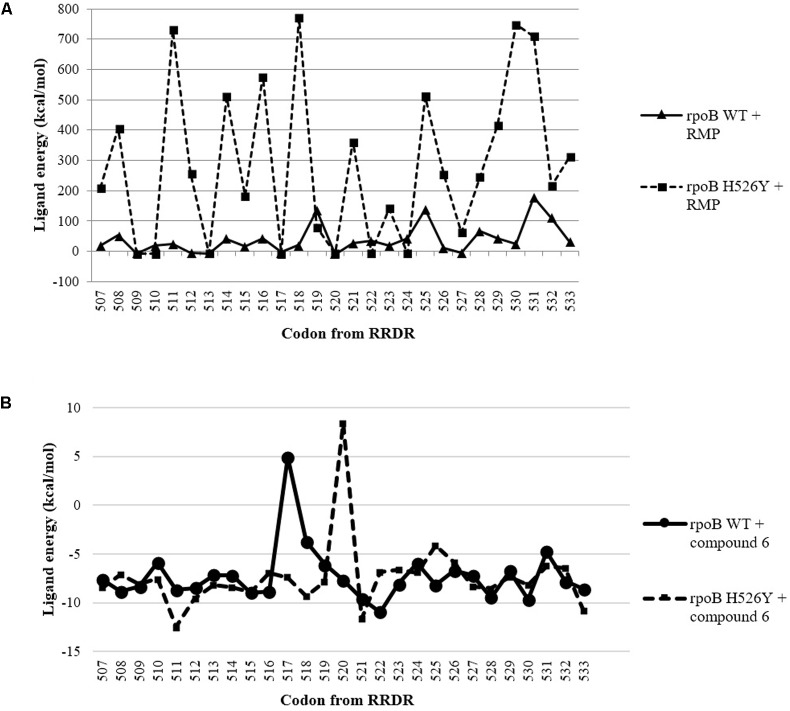
Ligand energy between each codon from RRDR of *Escherichia coli rpoB* gene (WT and H526T) and RMP **(A)**; and compound **6 (B)**.

The compound **6** was able to inhibit RMP_R_ strain (MIC = 12.5 μM) at a concentration 50x lower than the drug RMP (MIC = 622.2 uM) and, considering the peculiarity of this strain – which differs from the H37Rv mainly by the mutation in the *rpoB* gene (H526Y) – we were prompted to explore a possible affinity of this compound with mutant *rpoB*. By preliminary docking, we observed a reduction in the affinity between RMP and the RRDR site in mutant *rpoB* target (**Figure 1A**) while compound **6** showed more negative free energy – strong binding (Silva et al., 2017) – compared to RMP for both wild-type as well as the mutant protein (**Figure 1**).

Regarding the ADME analysis, all the naphthoquinones evaluated in this study showed high gastrointestinal absorption and are in agreement with the Lipinski’s rule-of-five (Lipinski, 2004): molecular weight ≤ 500, miLogP ≤ 5, H-bond donors ≤ 5, and H-bond acceptors ≤ 10 (**Table 3**), indicating crucial characteristics for oral bioavailability. In addition, most of the compounds showed none or low toxicity risk related to mutagenicity or tumorigenicity (**Table 3**).

**Table 3:** Absorption, distribution, metabolism, and excretion (ADME) characterization and toxicity risks of naphthoquinones.

Compound	MW	miLogP	TPSA (Å^2^)	H-Acceptors	H-Donors	GI absorption	Mutagenic	Tumorigenic
**1**	227.04	2.88	34.14	2	0	High	Low	High
**2**	242.27	2.95	43.38	3	0	High	None	None
**3**	228.24	2.43	43.38	3	0	High	None	None
**4**	214.22	2.30	43.38	3	0	High	Low	None
**5**	173.17	1.08	60.16	3	2	High	High	None
**6**	249.26	3.59	46.17	3	1	High	Low	None

*MW, molecular weight; miLogP, octanol–water partition coefficient logP; TPSA, total polar surface area; GI absorption, gastrointestinal absorption.*

In this study, by the pharmacokinetic analysis using the Software Swiss ADME, the theoretical inhibitory activity of naphthoquinone derivatives against five cytochrome P450 isoforms was evaluated (CYP450): CYP1A2, CYP2C9, CYP2C19, CYP2D6, and CYP3A4. All compounds showed potential inhibitory activity of CYP1A2 and CYP2C19 isoforms, except for compound **5**, which showed interaction only with CYP1A2. In addition, compounds **2** and **6** also showed activity against the 3A4 isoform.

## Discussion

We evaluated the antimycobacterial potential of six 1,4-naphthoquinone derivatives: all compounds were active against susceptible and resistant strains of *M. tuberculosis*, with different MIC values. Mathew et al. (2010) reported the antimycobacterial activity of plumbagin and its derivatives, and Dey et al. (2014) reported the susceptibility of mycobacteria (sensitive and resistant) to naphthoquinones, mainly plumbagin, with MIC between 0.25 and 16 μg/mL. Thus, both highlight the potential of naphthoquinones in the search for new anti-TB drugs. Our research group has developed several studies with a focus on new antimicrobial agents and, although other studies have reported the activity of other naphthoquinone derivatives, including for resistant strains, their mechanism of action is still uncertain (Coelho et al., 2010; Jardim et al., 2015).

Based on the analysis of the structure-activity relationship, the compound **3**, which has a tetrahydrofuran radical and anti-trypanosome activity previously described (De Moura et al., 2001), showed MIC = 54.8 μM against the three *M. tuberculosis* strains, while the compound **2**, which contains a tetrahydropyran radical, showed MIC between 103.3 and 206.6 μM. Besides showing a better antimycobacterial activity, the compound **3** has also shown reduced cytotoxicity (IC_50_ > 877 μM) compared with **2** (IC_50_ = 285 μM), and both showed none mutagenic or tumorigenic risks (**Table 3**).

When we analyzed the compounds with nitrogen (**5** and **6**), it was noticed that phenylamine radical in the compound **6** has decreased the activity for the susceptible and INH_R_ strains, while was able to a threefold increase the activity of this naphthoquinone for RMP_R_ strain, compared with compound **5**, which has the amine group (**Table 2**). The activity of naphthoquinones with nitrogenous radicals also has been described against fungi, gram positive and negative bacteria (Riffel et al., 2002; Rahmoun et al., 2013).

Among the rifampicin-resistant *M. tuberculosis*, about 35% have the mutation H526Y (Molodtsov et al., 2017) and, considering that compound **6** was active against the RMP_R_ strain, we can infer that there is probably no cross-resistance between this compound and RMP, what was also evidenced in the docking analysis (Molodtsov et al., 2017). Moreover, due to the lower binding energies of the compound **6** when compared to the binding energies of RIF in RRDR, the mutation seems to favor the binding between the naphthoquinone 6 and the RRDR (**Figure 1**).

Furthermore, among the naphthoquinone derivatives evaluated, the compound **5** was the most active for the INH_R_ strain (MIC = 36.1 μM), which has a mutation in the *katG* gene (S315T). This result evidences the absence of cross-resistance of this compound with INH, especially in strains that have this mutation.

According to the results of *in silico* pharmacokinetics, the naphthoquinone derivatives have inhibitory potential in some human CYP450 isoforms. It is known that the electron transfer chain is directly related with activity of enzymes, such as NADH, transferring electrons from lipophilic redox carriers. In this context, the importance of this biosynthetic route as a target in the search for new antimicrobials has been highlighted (Black et al., 2014) and, considering the results obtained in this study, the naphthoquinones could act as other quinones previously described, in the transfer chain of electrons (Heikal et al., 2016).

## Conclusion

Considering the potential of quinones as a source of new antimicrobial agents and their biological activities already reported, this set of findings confirm this hypothesis and demonstrate that, naphthoquinones derivatives could be scaffolds for the development of new anti-TB drugs.

## Author Contributions

PH, DR, and PdS designed the study. KDM, PC, KDR, TC, and MP contributed with compounds synthesis. PH, LF, and DR performed the experiments. PH, DR, PdS, and KDM analyzed the data and wrote the manuscript. All authors approved the version to be published.

## Conflict of Interest Statement

The authors declare that the research was conducted in the absence of any commercial or financial relationships that could be construed as a potential conflict of interest.

## Footnotes

1 molinspiration.com/cgi-bin/properties

2 http://www.swissadme.ch/index.php

3 www.organic-chemistry.org/prog/peo/

4 https://www.rcsb.org
